# Impact of Laparoscopic Gastrectomy on the Completion Rate of the Perioperative Chemotherapy Regimen in Gastric Cancer: A Swedish Nationwide Study

**DOI:** 10.1245/s10434-023-13967-6

**Published:** 2023-07-28

**Authors:** Andrianos Tsekrekos, David Borg, Victor Johansson, Magnus Nilsson, Fredrik Klevebro, Lars Lundell, Maria Gustafsson-Liljefors, Ioannis Rouvelas

**Affiliations:** 1 Department of Upper Abdominal Diseases, Karolinska University Hospital C1:77, Stockholm, Sweden; 2 Division of Surgery and Oncology, Department of Clinical Science, Intervention and Technology (CLINTEC), Karolinska Institutet, Stockholm, Sweden; 3 Oncology Department, Skåne University Hospital, Lund, Sweden; 4 Division of Oncology and Therapeutic Pathology, Department of Clinical Sciences, Lund University, Lund, Sweden; 5 Department of Oncology, Sahlgrenska University Hospital, Gothenburg, Sweden; 6 Department of Surgery, Odense University Hospital, Odense, Denmark

## Abstract

**Background:**

Omission of prescheduled chemotherapy following surgery for gastric cancer is a frequent clinical problem. This study examined whether laparoscopic gastrectomy (LG) had a positive impact on compliance with adjuvant chemotherapy compared with open (OG).

**Methods:**

Patients with cT2-4aN0-3M0 adenocarcinoma treated with gastrectomy and perioperative chemotherapy between 2015 and 2020 were identified in the Swedish national register. Additional information regarding chemotherapy was retrieved from medical records. Regression models were used to investigate the association between surgical approach and the following outcomes: initiation of adjuvant chemotherapy, modification, and time interval from surgery to start of treatment.

**Results:**

A total of 247 patients were included (121 OG and 126 LG, conversion rate 11%), of which 71.3% had performance status ECOG 0 and 77.7% clinical stage II/III. In total, 86.2% of patients started adjuvant chemotherapy, with no significant difference between the groups (LG 88.1% vs OG 84.3%, *p* = 0.5). Reduction of chemotherapy occurred in 37.4% of patients and was similar between groups (LG 39.4% vs OG 35.1%, *p* = 0.6), as was the time interval from surgery. In multivariable analysis, LG was not associated with the probability of starting adjuvant chemotherapy (OR 1.36, *p* = 0.4) or the need for reduction (OR 1.29, *p* = 0.4). Conversely, major complications had a significant, negative impact on both outcomes.

**Conclusions:**

This nationwide study demonstrated a high rate of adjuvant chemotherapy initiation after curative intended surgery for gastric cancer. A beneficial effect of LG compared with OG on the completion rate was not evident.

**Supplementary Information:**

The online version contains supplementary material available at 10.1245/s10434-023-13967-6.

The majority of patients diagnosed with gastric cancer (GC) in Europe present with advanced tumors and their prognosis is poor, with cure achieved only in approximately 25% of cases.^1,2^ The fact that many patients relapse following a radical—and potentially curative—resection suggests that non-detectable metastatic lesions were already present at the time of surgery. Multimodal treatment strategies have evolved to improve survival, mainly by combining surgery with systemic treatment in the form of perioperative (neoadjuvant and adjuvant) chemotherapy. Downstaging of the primary tumor and elimination of occult micro-metastases constitute the main rationale for adding chemotherapy whenever feasible. European trials have demonstrated the survival benefit of this approach^3–5^, which is currently considered the standard of care.^6^

The chemotherapy regimens used in GC have a postoperative component; however, it is not uncommon that patients are unable to receive chemotherapy after surgery due to surgical morbidity, poor general condition, compromised nutritional status, or treatment-related toxicity. This has been a consistent finding in all three aforementioned randomized trials; in the MAGIC^3^, the FNCLCC/FFCD 9703^4^, and most recently the FLOT4-AIO trial^5^, half of the patients did not start or discontinued the scheduled adjuvant chemotherapy.

An important step in the evolution of the treatment for GC has been the introduction of minimally invasive surgery. In recent years, several RCTs and population-based studies have compared laparoscopic gastrectomy (LG) with open gastrectomy (OG) with regard to short-term surgical outcomes (postoperative complications and mortality, functional recovery, length of hospital stay),^7–10^ oncological safety (radicality, number of resected lymph nodes) and long-term survival.^11–15^ Notably, the issue of whether the advantage of a more favorable postoperative course after LG has a positive impact on compliance with the ensuing chemotherapy has not been thoroughly investigated, even though intolerance for, and eventual omission of the adjuvant chemotherapy is a frequent clinical problem. Given that LG is less invasive and has been shown to reduce morbidity and enhance patient recovery, its wider application might result in a higher completion rate of the adjuvant treatment. Evidence is still limited on this topic and the available results are inconsistent, as a few retrospective studies have suggested a possible advantage of LG,^14,16,17^ while others have not shown any difference related to the surgical approach.^18,19^

The aim of this nationwide study was to examine whether the choice between LG and OG affects the completion rate of perioperative chemotherapy regimens in terms of rate of initiation, need for modification, and time interval to start of chemotherapy after surgery for GC.

## Methods

This study is reported following the Strengthening the Reporting of Observational Studies in Epidemiology (STROBE) recommendations.^20^ Ethical approval was obtained from the Regional Research Ethics Committee of Stockholm (2013/596-31/3, 2016/1486-32, and 2020-06495). The complementary review of medical records was approved by the institutional review board at each participating hospital, or the regional authority legally responsible for the handling of personal data, in compliance with the General Data Protection Regulation (GDPR) requirements.

### Data Source

Eligible patients were identified in the Swedish National Register for Esophageal and Gastric Cancer (NREV).^21^ This national quality register prospectively collects data on patients diagnosed with these malignancies in Sweden and has been described in detail elsewhere.^22^ Data are acquired through a number of surveys at different time points, reported on a web module by the hospital responsible for the treatment and follow-up of the patient. The NREV database has previously been validated and shown to have a high grade of data completeness and accuracy.^23^ The register was the source of information on patient demographics, tumor characteristics, details of the surgical treatment, and postoperative outcomes, including histopathological findings. Since the survey concerning the details of the oncological treatment was introduced in 2017, and thus not available for the entire study period, information regarding the neoadjuvant and adjuvant chemotherapy was retrieved by reviewing the patients’ medical records. This included type of chemotherapy regimen, time interval between surgery and initiation of chemotherapy, number of cycles initially planned and ultimately administered, regimen modifications, and reason for not initiating the adjuvant treatment. During this review process, missing register data (mainly owing to unreported follow-up surveys) were also added.

### Study Population

Patients treated with curative intent for adenocarcinoma of the stomach or gastroesophageal junction Siewert type III^24^ between 1 January 2015 and 31 December 2020 were identified in NREV. The study inclusion criteria were: (1) clinical stage T2-4aN0-3M0 according to the 8th edition of the Union for International Cancer Control (UICC) *TNM Classification of Malignant Tumors*,^25^ (2) combined modality treatment, consisting of surgery and perioperative chemotherapy, (3) Standard open or laparoscopic gastrectomy performed (distal or total gastrectomy, with or without splenectomy),^26^ and (4) No other concomitant malignancy.

### Exposure

Included patients were stratified into two groups according to surgical approach, i.e., LG and OG (reference group). Analyses were performed based on the intention-to-treat principle, with laparoscopic procedures converted to open included in the LG group.

### Outcome Measures

Regression models were applied to investigate the impact of the surgical approach on the following three outcomes: (1) start of adjuvant chemotherapy (yes/no), (2) time interval between surgery and start of adjuvant chemotherapy (days), and (3) overall reduction of adjuvant chemotherapy (yes/no). The latter was defined as the occurrence of one of the following: (a) modification of the adjuvant chemotherapy regimen compared with the neoadjuvant with regard to the number of chemotherapy agents (i.e., reduction from triplet to doublet regimen, or from doublet regimen to monotherapy), or (b) a similar reduction in the number of chemotherapy agents during the course of the adjuvant treatment, or (c) reduction of the number of courses of the prescheduled adjuvant chemotherapy, i.e., a premature termination of the adjuvant treatment.

### Statistical Analysis

All statistical analyses were performed using R statistical software version 4.2.1 (R Foundation for Statistical Computing, Vienna, Austria).^27^ Statistical significance was defined as *p*-value < 0.05.

Multivariable logistic regression models were fitted to the data to assess the impact of the exposure of main interest on outcomes (1) and (3) defined above, with strength of association expressed as odds ratio (OR) with corresponding 95% confidence interval (CI). A multivariable linear regression model with logarithmic transformation (natural logarithm) was applied for outcome (2). All models incorporated the following five predetermined predictors: age (years, continuous), sex, Eastern Cooperative Oncology Group (ECOG) performance status (0 vs 1–2), clinical stage according to the UICC TNM classification (ordinal I, II, III),^25^ and occurrence of major postoperative complications, defined as grade ≥ IIIa according to Clavien-Dindo (CD).^28^ Residuals were analyzed to check whether the assumptions of linearity, normality, and homoscedasticity were met. Outliers and influential values were visualized with diagnostic plots, and goodness of fit was evaluated for the linear and logistic regression models (adjusted *R*^2^ and Hosmer-Lemeshow statistic, respectively).

## Results

**Fig. 1:**
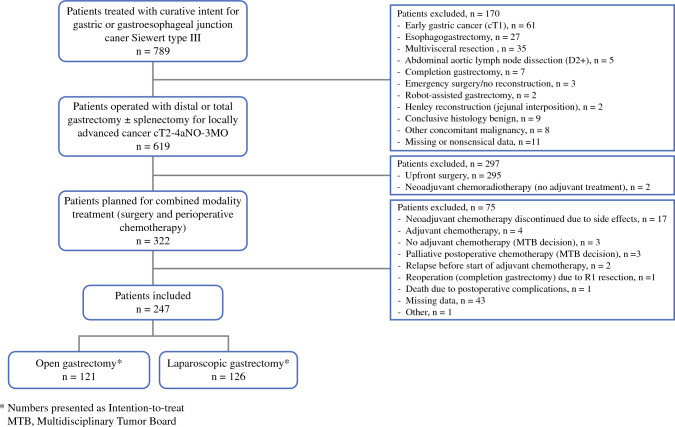
Flow chart of the selection of the study population

A total of 789 potentially eligible patients were identified, of which 322 (41%) met the inclusion criteria. Review of the medical records was possible for 279 (87%) of these patients, treated in 20 Swedish hospitals (Supplementary Table 1). After exclusion of another 32 patients following review of their records, 247 patients remained and were eligible for analysis. A flow chart of the selection of the study population, with reasons for patient exclusion, is provided in Fig. 1.

### Demographics, Disease Characteristics, and Surgical Outcomes

Demographics and tumor characteristics of the cohort are summarized in Table 1. The median age of the study population was 65 years (range 26 to 82 years) and of the 247 patients included, 99 (40.1%) were female. With regard to performance status, 71.3% of the patients were classified as ECOG 0 and 28.7% as ECOG 1 or 2. The distribution of clinical disease stage in the cohort was 22.3% stage I, 46.9% stage II, and 30.8% stage III. The treatment groups were balanced at baseline, with the exception of tumor location. More specifically, the LG group included a higher proportion of patients with distal tumors (pylorus and antrum), while proximal tumors (corpus, fundus, and cardia type III) were more common in the OG group. However, with regard to the extent of resection (distal or total gastrectomy), there was no significant difference between the groups (total gastrectomy, LG 49.2% vs OG 56.2%, *p* = 0.3).

**Table 1:** Patient demographics and tumor characteristics by surgical approach

	Total	OG	LG	p*
	*n* = 247	*n* = 121	*n* = 126	
Age, median (IQR)	65 (56, 71)	68 (56, 72)	64 (55, 70)	0.08
BMI, median (IQR)	25.4 (23.1, 28.8)	25.1 (23.1, 28.9)	25.8 (23.2, 28.4)	0.8
*Sex*				0.3
Male	148 (59.9)	68 (56.2)	80 (63.5)	
Female	99 (40.1)	53 (43.8)	46 (36.5)	
*ECOG performance status*				> 0.9
0	176 (71.3)	86 (71.1)	90 (71.4)	
1-2	71 (28.7)	35 (28.9)	36 (28.6)	
*Clinical T category*				0.7
T2	62 (25.1)	29 (24.0)	33 (26.2)	
T3	140 (56.7)	72 (59.5)	68 (54.0)	
T4a	45 (18.2)	20 (16.5)	25 (19.8)	
*Clinical N category*				0.4
N0	164 (66.4)	80 (66.1)	84 (66.7)	
N1	45 (18.2)	26 (21.5)	19 (15.1)	
N2	28 (11.3)	11 (9.1)	17 (13.5)	
N3	10 (4.1)	4 (3.3)	6 (4.8)	
*Clinical TNM stage*				0.3
I	55 (22.3)	23 (19.0)	32 (25.4)	
II	116 (46.9)	63 (52.1)	53 (42.1)	
III	76 (30.8)	35 (28.9)	41 (32.5)	
*Tumor location*				**0.037**
Pylorus	26 (10.5)	12 (9.9)	14 (11.1)	
Antrum	88 (35.6)	33 (27.3)	55 (43.6)	
Corpus	83 (33.6)	45 (37.2)	38 (30.2)	
Fundus	21 (8.5)	13 (10.7)	8 (6.3)	
Cardia type III	21 (8.5)	15 (12.4)	6 (4.8)	
Multifocal or unspecified	8 (3.2)	3 (2.5)	5 (4.0)	

Data presented as n (%), unless otherwise indicated. Percentages may not add up to 100% because of rounding.

*OG* open gastrectomy, *LG* laparoscopic gastrectomy, *IQR* interquartile range, *BMI* body mass index, *ECOG* Eastern Cooperative Oncology Group.

*Wilcoxon rank sum test for continuous variables. Pearson’s *χ*^2^ test for categorical variables (Fisher’s exact test for categorical variables with any expected cell count < 5). Significant values (*p* < 0.05) are indicated with bold type.

Postoperative outcomes, including histopathological findings, are presented in Table 2. One hundred and twenty-one patients underwent OG and 126 patients LG, of which 14 (11.1%) were converted to open surgery. There was no difference in the rate of overall postoperative complications (CD grade ≥ II, LG 34.9% vs OG 38.0%, *p* = 0.7), or the occurrence of major complications (CD grade ≥ IIIa, 19.8% in both groups, *p* >0.9). The groups were also comparable regarding ypTNM stage, microscopically tumor-free resection margins (LG 95.2% vs OG 90.1%, *p* = 0.14), and rate of complete tumor regression (LG 6.4% vs OG 10.7%, *p* = 0.3). The only difference observed concerned the median number of resected lymph nodes, which was significantly higher in the LG group (34 vs 27, *p* = 0.006).

**Table 2:** Type of surgical procedure and postoperative outcomes

	Total	OG	LG	p*
	*n* = 247	*n* = 121	*n* = 126	
Conversion to open surgery	–	–	14 (11.1)	
*Type of gastrectomy*				0.3
Total	130 (52.6)	68 (56.2)	62 (49.2)	
Distal	117 (47.4)	53 (43.8)	64 (50.8)	
Overall complications—CD grade ≥ II	90 (36.4)	46 (38.0)	44 (34.9)	0.7
Major complications—CD grade ≥ IIIa	49 (19.8)	24 (19.8)	25 (19.8)	> 0.9
*ypT stage*				0.6
T0	21 (8.5)	13 (10.7)	8 (6.4)	
T1/HGD	44 (17.8)	18 (14.9)	26 (20.6)	
T2	40 (16.2)	20 (16.5)	20 (15.9)	
T3	80 (32.4)	38 (31.4)	42 (33.3)	
T4	62 (25.1)	32 (26.5)	30 (23.8)	
*ypN stage*				0.088
N0	120 (48.6)	49 (40.5)	71 (56.3)	
N1	42 (17.0)	23 (19.0)	19 (15.1)	
N2	47 (19.0)	26 (21.5)	21 (16.7)	
N3	38 (15.4)	23 (19.0)	15 (11.9)	
*ypM stage*				> 0.9
M0	240 (97.2)	118 (97.5)	122 (96.8)	
M1	7 (2.8)	3 (2.5)	4 (3.2)	
Complete tumor regression	21 (8.5)	13 (10.7)	8 (6.4)	0.3
Resected lymph nodes, median (IQR)	28 (21, 41)	27 (20, 35)	34 (23, 42)	**0.006**
*Radicality*				0.14
R0	229 (92.7)	109 (90.1)	120 (95.2)	
R1	18 (7.3)	12 (9.9)	6 (4.8)	

Data presented as n (%), unless otherwise indicated. Percentages may not add up to 100% because of rounding.

*OG* open gastrectomy, *LG* laparoscopic gastrectomy, *CD* Clavien-Dindo, *IQR* interquartile range, *HGD* high-grade dysplasia

*Wilcoxon rank sum test for continuous variables. Pearson’s *χ*^2^ test for categorical variables (Fisher’s exact test for categorical variables with any expected cell count < 5). Significant values (*p* <0.05) are indicated with bold type.

### Start of Adjuvant Chemotherapy

**Fig. 2:**
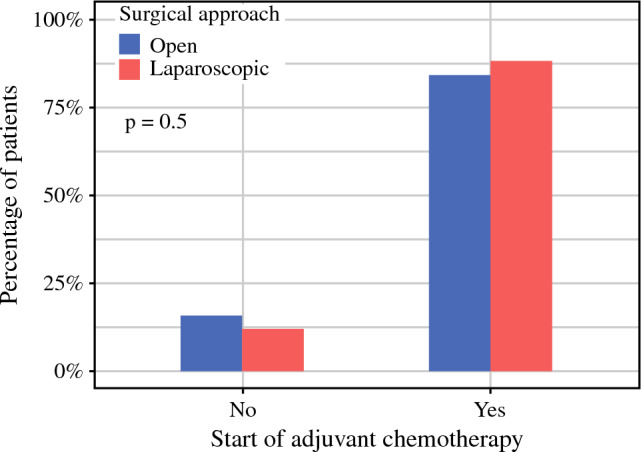
Proportion of patients starting adjuvant chemotherapy

Of the 247 patients included in the study, 213 patients (86.2%) started adjuvant chemotherapy, with no significant difference between the groups (LG 88.1% vs OG 84.3%, *p* = 0.5, Fig. 2). Thirty-four patients (13.8%) did not receive adjuvant treatment due to one, or a combination, of the following reasons: (1) poor general condition, (2) compromised nutritional status, (3) surgical morbidity, (4) COVID-19 infection, or (5) patient decision. The reason for not proceeding with adjuvant chemotherapy as initially planned was not specified in 6 cases.

Multivariable logistic regression showed that the surgical approach was not associated with the probability of starting chemotherapy after surgery (OR 1.36, 95% CI 0.62–3.01, *p* = 0.4). Of the covariates included in the model, only the occurrence of major complications was shown to have a significant, negative impact on this outcome (OR 0.28, 95% CI 0.12–0.62, *p* = 0.002).

### Time from Surgery to Start of Adjuvant Chemotherapy

**Fig. 3:**
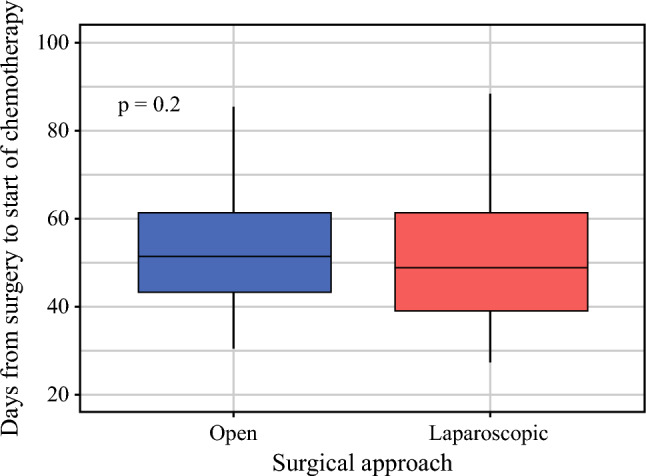
Days from surgery to start of chemotherapy. The thick lines in the middle correspond to median values. The lower and upper horizontal lines correspond to the first and third quartiles (the 25th and 75th percentiles). The whiskers extend to the highest and lowest values, at most 1.5 x IQR (interquartile range)

In the subset of patients that started adjuvant chemotherapy (n = 213), the time interval from surgery was slightly shorter following LG compared with OG, but this difference was not statistically significant (median 49 days [IQR 39, 62] vs 51 days [IQR 43, 61], *p* = 0.2, Fig. 3). Multivariable linear regression did not reveal any independent variable that significantly influenced the time to start of adjuvant chemotherapy after gastrectomy (analysis not shown).

### Overall Reduction of Adjuvant Chemotherapy

**Fig. 4:**
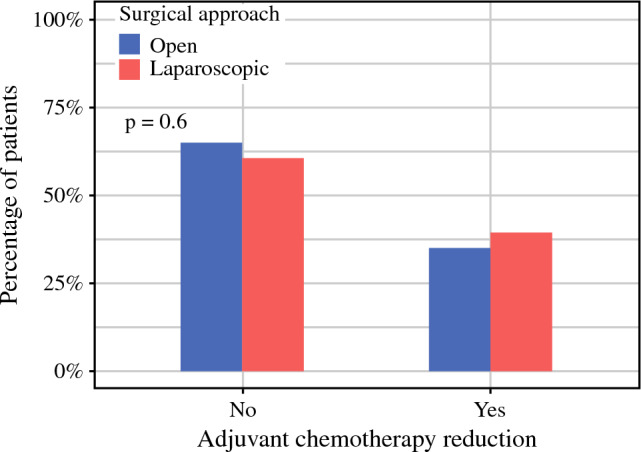
Proportion of patients receiving reduced adjuvant chemotherapy

Evaluation of any reduction of chemotherapy following gastrectomy was irrelevant or not possible for 15 of the 213 patients (7%). The majority were either patients that relapsed during the course of the adjuvant treatment, leading to shift of the chemotherapy regimen to a palliative alternative, or patients that received more chemotherapy postoperatively compared with before surgery, due to tumor-related symptoms (mainly gastric outlet obstruction). In the remaining study population (n = 198), reduction of chemotherapy occurred in 74 patients in total (37.4%), with the proportion of patients receiving reduced chemotherapy being comparable between the two exposure groups (LG 39.4% vs OG 35.1%, *p* = 0.6) (Fig. 4).

Multivariable logistic regression did not show any association between the surgical approach and the need to reduce the adjuvant chemotherapy (OR 1.29, 95% CI 0.71–2.36, *p* = 0.4). Conversely, the occurrence of major postoperative complications was associated with a higher probability of receiving reduced chemotherapy after the operation (OR 2.15, 95% CI 1.00–4.65, *p* = 0.049). As for the remaining covariates in the model, greater age also significantly increased the odds of chemotherapy reduction after surgery (OR 1.04, 95% CI 1.01–1.07, *p* = 0.012).

## Discussion

In this nationwide study, we investigated the impact of a minimally invasive surgical approach on different aspects of the completion of perioperative chemotherapy in GC. After adjusting for clinically relevant covariates, no association between the surgical approach and the rate of initiation, the need for regimen modification, or the time interval to start of the adjuvant chemotherapy became evident. Of the covariates included in the regression models, the occurrence of major postoperative complications was found to be an independent and strong predictor of omission of the adjuvant treatment. In addition, severe morbidity was also associated with a higher rate of chemotherapy reduction following surgery.

Whether the neoadjuvant and adjuvant parts of perioperative chemotherapy regimens are equally important for long-term prognosis is not yet clarified. Asian RCTs have clearly demonstrated the survival benefit of adjuvant chemotherapy in patients with stage II/III gastric cancer that have undergone up-front curative resection.^29–31^ In Europe, however, the preferred modality is perioperative chemotherapy, meaning that patients are already exposed to chemotherapy before surgery. Although evidence is limited, there are studies indicating that the adjuvant portion of chemotherapy may still be of value also in this setting. Glatz et al. studied 134 patients with esophagogastric cancer and found that postoperative chemotherapy contributed substantially to the beneficial effect of the perioperative regimen on survival.^32^ Similarly, Saunders et al. analyzed 333 patients and showed that the administration of adjuvant chemotherapy conferred a significant survival benefit, although this was limited to patients with proven histopathological response to the neoadjuvant treatment.^33^ Although both studies have certain limitations (retrospective, single center studies with predominantly cardia tumors), they imply that the adjuvant part of perioperative chemotherapy regimens plays a role in recurrence prevention. Nevertheless, the fact that approximately half of the patients will eventually not proceed with adjuvant chemotherapy as intended strengthens the argument that the preoperative component is the most crucial, either as the neoadjuvant part of a perioperative scheme, or as a totally neoadjuvant approach (usually in the form of chemoradiotherapy and mainly for tumors located at the gastroesophageal junction). This not only ensures delivery of systemic treatment when it is most likely that the patient will be able to tolerate it, but also provides an observation period during which subclinical dissemination may manifest, and thus spare some patients with biologically aggressive tumors a futile procedure.

A recent population-based study by our group comparing LG and OG for advanced gastric cancer implied that LG could be safely preformed, with acceptable morbidity and mortality, and was associated with improved overall survival compared with OG.^15^ The results of the current study do not support our hypothesis that a minimally invasive surgical approach may lead to a higher rate of administration of the adjuvant treatment; LG was related to an adjusted OR of 1.36 which was not statistically significant. Thus, we could not confirm the exploratory findings of other Western studies, e.g., a case-control study from the Memorial Sloan-Kettering Cancer Center^16^ and a multicenter study conducted by the Italian Research Group for Gastric Cancer.^14^ Those studies also included patients treated with neoadjuvant chemotherapy and implied a possible advantage of LG with regard to the likelihood of proceeding with the intended adjuvant treatment. Our results are instead in agreement with a large study derived from the U.S. National Cancer Database, where the proportion of patients receiving adjuvant chemotherapy did not differ between LG and OG.^19^ On the other hand, it is noteworthy that in the current study an exceptionally higher proportion of patients (86.2%) proceeded with adjuvant chemotherapy, compared with rates reported in previous studies. Thus, a potential true association with surgical approach would have been difficult to demonstrate and would require a much larger sample.

Studies from high-incidence countries have also shown that besides initiation, compliance with adjuvant chemotherapy, as well as its duration—which corresponds to the cumulative dose administered—have an impact on survival.^34–36^ Still, modifications of the chemotherapy regimen, such as reductions in the dosage or the number of chemotherapy agents compared with the initial scheme, delays in the prescheduled cycles, and even definite withdrawal from further oncological treatment, are not uncommon following a highly invasive procedure like gastrectomy. A phase II RCT designed to explore the safety of LG after neoadjuvant chemotherapy also demonstrated that patients in the laparoscopic group could better tolerate the delivered adjuvant chemotherapy.^37^ In our study, the odds of postoperative chemotherapy reduction was similar between the groups.

In contrast to the negative results when assessing the effect of surgical approach, our models identified the occurrence of major postoperative complications as an independent predictor with significant impact on both outcomes: patients experiencing major complications were not able to start adjuvant treatment to the same extent as patients that had an uneventful recovery (adjusted OR 0.28, *p* = 0.002), and were also more likely to require a reduction of the chemotherapy regimen at some point (adjusted OR 2.15, *p* = 0.049). The importance of an uncomplicated postoperative course for the completion of the oncological treatment is well documented.^38–40^ Jin et al. explored the combined effect of complications and failure to receive adjuvant therapy on survival and found a significant interaction, leading to increased hazard of death by an estimated 130%. Interestingly, they also showed that, while postoperative morbidity had an independently negative impact on survival, that effect became non-significant among patients who actually managed to receive adjuvant treatment.^40^ The observed relationship between morbidity and omitted adjuvant chemotherapy has been suggested as a possible mechanism to explain the worse prognosis generally linked to postoperative complications.

The clinical importance of timing of adjuvant chemotherapy and its influence on prognosis and survival is debated, and published results have been contradictive. Park et al. studied a cohort of 840 GC patients and found that delay of adjuvant chemotherapy for longer than 8 weeks was associated with worse relapse-free and overall survival.^41^ Other studies, considering shorter time intervals between surgery and start of chemotherapy, such as 6^42^ or even 4 weeks,^43^ have come to the same conclusion. Conversely, there have been several reports that delay in starting systemic treatment, sometimes for as long as 12 weeks after surgery, did not have a negative prognostic impact.^35,44,45^ Nevertheless, it is important to note that all those studies excluded patients that had received neoadjuvant chemotherapy. A Dutch population-based study including 463 patients treated with perioperative chemotherapy and gastrectomy demonstrated that the time interval to start of adjuvant chemotherapy did not influence overall survival, given that it was within 12 weeks after surgery.^46^ Some of the aforementioned Western studies have examined whether the surgical approach affects this time interval, without showing any difference between LG and OG.^9,14,19^ This was confirmed by our data, where we found an approximately 7-week interval in both treatment groups. On the other hand, a retrospective study from Japan specifically addressing this question demonstrated a significant, but modest, median difference of 1 week in favor of LG.^18^ We believe that, even if future studies confirm that this is the case, differences of that magnitude have no relevance from a clinical point of view and should not alter patient prognosis.

Our study has limitations related to the non-randomized, retrospective nature of observational studies in general. Even though we adjusted for several clinicopathological factors, our results may be subject to residual confounding. One issue that especially deserves to be mentioned is that the information on performance status is registered adjacent to diagnosis, i.e., prior to the start of any treatment. Thus, it does not reflect the patient’s actual general condition before the start of adjuvant treatment, which undoubtedly could be affected by the preceding neoadjuvant chemotherapy and surgery. Furthermore, despite the nationwide design, the number of included patients may not be large enough to detect small, though significant, differences between the groups. Of note, more sophisticated methods for assessing the intensity of chemotherapy are in use, e.g., calculation of the Relative Dose Intensity (RDI), which takes into account both dose reductions and treatment delays.^47^ This requires detailed data on exact dose for each chemotherapy agent, date of delivery, and patient weight on the day of treatment, which was not available for many patients in our cohort. This was a multicenter study, including both teaching and county hospitals from the entire country, probably leading to a high grade of inter-institutional heterogeneity as reflected by the considerable variability in the chemotherapy protocols (Supplementary Table 2). This is at the same time one of the strengths of this study, i.e., the participation of the majority of hospitals that treat patients with GC in Sweden, providing real-world data. Since the patients included comprise 87% of the potentially eligible patients that were identified in the national register, our cohort provides a reliable representation of the Swedish population. Thus, it is fair to assume that these findings reflect the current practice with regard to perioperative chemotherapy for GC in Sweden, as well as the actual impact of the laparoscopic approach on the completion rate of the perioperative chemotherapy regimens.

## Conclusions

This study demonstrated a high initiation rate of adjuvant chemotherapy after curative intended surgery for GC in Sweden. The results could not show a beneficial effect of LG on the completion rate of perioperative chemotherapy regimens in terms of rate of initiation, need for modification, or time to start of adjuvant chemotherapy. Major postoperative complications were strongly associated with omission of adjuvant chemotherapy and an increased likelihood of chemotherapy reduction, which occurred in 44% of patients. These results confirm that complications are a crucial determinant of the ability to proceed with oncological treatment. Given that morbidity rates as high as 40% are reported in the West, multidisciplinary interventions in perioperative care should focus on minimizing complications, ultimately increasing the rates of adjuvant therapy completion and presumably leading to improved prognosis.

### Supplementary Information

Below is the link to the electronic supplementary material.

Supplementary file1 (DOCX 23 kb)

Supplementary file2 (DOCX 40 kb)
