# Supplementary material for: Impact of Laparoscopic Gastrectomy on the Completion Rate of the Perioperative Chemotherapy Regimen in Gastric Cancer: A Swedish Nationwide Study
Source: Ann Surg Oncol. 2023 Jul 28;30(12):7196–205. doi: 10.1245/s10434-023-13967-6 (PMC10562295; doi:10.1245/s10434-023-13967-6)
Supplement: Supplementary file 1 — Supplementary file1 (DOCX 23 kb) [file 10434_2023_13967_MOESM1_ESM.docx]

| **SUPPLEMENTARY TABLE 1**  List of hospitals participating in the study | |
| --- | --- |
| Danderyds Hospital | Sahlgrenska University Hospital |
| Eskilstuna Hospital | Skåne University Hospital |
| Gävle Hospital | Sunderby Hospital |
| Helsingborg Hospital | Söder Hospital Stockholm |
| Kalmar County Hospital | Södra Älvsborg Hospital |
| Karlstad Central Hospital | Uddevalla Hospital |
| Karolinska University Hospital | University Hospital of Umeå |
| Kristianstad Central Hospital | Uppsala University Hospital |
| Linköping University Hospital | Växjö Central Hospital |
| Ryhov Hospital Jönköping | Örebro University Hospital |
