# Supplementary material for: Impact of Laparoscopic Gastrectomy on the Completion Rate of the Perioperative Chemotherapy Regimen in Gastric Cancer: A Swedish Nationwide Study
Source: Ann Surg Oncol. 2023 Jul 28;30(12):7196–205. doi: 10.1245/s10434-023-13967-6 (PMC10562295; doi:10.1245/s10434-023-13967-6)
Supplement: Supplementary file 2 — Supplementary file2 (DOCX 40 kb) [file 10434_2023_13967_MOESM2_ESM.docx]

| **SUPPLEMENTARY TABLE 2**  Summary of the different chemotherapy regimens used by the participating hospitals | | |
| --- | --- | --- |
| **Regimen** | **Neoadjuvant (n = 247)** | **Adjuvant (n = 213)** |
| Triplet | | |
| FLOT | 125 (50.6) | 101 (47.4) |
| EOX/EOF | 67 (27.1) | 46 (21.6) |
| FOLFOXIRI | 3 (1.2) | 2 (0.9) |
| FLv-Doc-Oxa | 3 (1.2) | 5 (2.4) |
| Doublet | | |
| FOLFOX | 22 (8.9) | 21 (9.9) |
| FLOX | 17 (6.9) | 13 (6.1) |
| XELOX | 5 (2.0) | 4 (1.9) |
| FOLFIRI | 4 (1.6) | 4 (1.9) |
| FLIRI | 1 (0.4) | 2 (0.9) |
| Doc-FLv-mdg | - | 2 (0.9) |
| SOX | - | 1 (0.5) |
| Monotherapy | | |
| FLv | - | 6 (2.8) |
| FLv-mdg | - | 4 (1.9) |
| 5-FU infusion | - | 2 (0.9) |

Data presented as n (%). Percentages may not add up to 100% because of rounding.

All regimens with 5-fluorouracil also contain folinic acid, except for EOF.

FLOT: 5-fluorouracil (infusion), oxaliplatin and docetaxel

EOX: epirubicin, oxaliplatin and capecitabine

EOF: epirubicin, oxaliplatin and 5-fluorouracil (infusion)

FOLFOXIRI: 5-fluorouracil (infusion), oxaliplatin and irinotecan

FLv-Doc-Oxa: 5-fluorouracil (bolus), docetaxel and oxaliplatin

FOLFOX: 5-fluorouracil (bolus plus infusion) and oxaliplatin

FLOX: 5-fluorouracil (bolus) and oxaliplatin

XELOX: capecitabine and oxaliplatin

FOLFIRI: 5-fluorouracil (infusion) and irinotecan

FLIRI: 5-fluorouracil (bolus) and irinotecan

Doc-FLv-mdg: docetaxel and 5-fluorouracil (modified de Gramont)

SOX: S-1 and oxaliplatin

FLv: 5-fluorouracil (bolus)

FLv-mdg: 5-fluorouracil (modified de Gramont)

5-FU: 5-fluorouracil
